# Advances in Endoscopic Ultrasound in Pancreatic Cancer Screening, Diagnosis, and Palliative Care

**DOI:** 10.3390/biomedicines13010076

**Published:** 2024-12-31

**Authors:** Wenyu Zhang, Jingzheng Chen, Wei Zhang, Min Xu

**Affiliations:** 1 Department of Gastroenterology, Affiliated Hospital of Jiangsu University, Zhenjiang 212001, China; 2 Department of Cardiology, Affiliated Hospital of Jiangsu University, Zhenjiang 212001, China; 3 Department of Gastroenterology, Digestive Disease Institute of Jiangsu University, Affiliated Hospital of Jiangsu University, Zhenjiang 212001, China

**Keywords:** endoscopic ultrasound, pancreatic cancer, early screening, palliative care, diagnosis

## Abstract

Pancreatic cancer is a highly aggressive malignancy with a profoundly poor prognosis. Clinically, the condition most frequently manifests with symptoms including painless jaundice, abdominal discomfort, and back pain. Early diagnosis and the implementation of effective therapeutic strategies are critical for improving patient survival outcomes. However, merely 10–20% of patients are diagnosed at an early stage, with the majority presenting at advanced stages, often with metastasis. Consequently, early detection and intervention are crucial for enhancing prognosis. The widespread adoption of endoscopic ultrasonography (EUS) technology in recent years has significantly enhanced the diagnostic accuracy for pancreatic space-occupying lesions. EUS is increasingly recognized for its pivotal role in alleviating malignant biliary obstruction (MBO), gastric outlet obstruction (GOO), and refractory pain in advanced pancreatic cancer. This article aims to provide an overall review of the current applications of EUS in the diagnosis and treatment of pancreatic cancer, exploring its advantages and limitations in early screening, diagnosis, and palliative care. Furthermore, this review explores potential future directions in the field, aiming to provide valuable insights to inform and enhance the clinical management of pancreatic cancer.

## 1. Introduction

Pancreatic cancer stands as the most aggressive malignancy among gastrointestinal tumors, marked by an exceedingly poor prognosis and a five-year survival rate of less than 9% [1,2,3]. In the absence of targeted and effective treatments, pancreatic cancer is expected to become the second leading cause of cancer-related deaths by 2030 [4]. The predominant risk factors associated with pancreatic cancer encompass dietary patterns characterized by poor nutritional quality, advanced age, tobacco use, excessive alcohol intake, a familial predisposition to the disease, and the presence of chronic pancreatitis [5,6]. Recent clinical investigations have established diabetes and hyperglycemia as critical risk factors for pancreatic cancer [7,8], with evidence indicating that individuals with type 2 diabetes exhibit nearly a twofold increased risk of developing pancreatic cancer compared to the general population [9]. Furthermore, chronic cholecystitis, a history of cholecystectomy, and reduced levels of high-density lipoprotein (HDL) cholesterol have also been recognized as significant risk factors contributing to the development of pancreatic cancer [10]. The targeted screening of people with risk factors can improve early diagnosis [11].

The predominant phenotype of pancreatic cancer is pancreatic ductal adenocarcinoma (PDAC), which accounts for over 85% of exocrine pancreatic malignancies [12,13]. When pancreatic cancer tumors are confined to the ductal epithelium and measure less than 1 cm in size, the five-year survival rate following treatment can approach 100% [14]. However, most pancreatic cancer patients present with nonspecific early symptoms, including fatigue, weight loss, and loss of appetite, which frequently lead to missed opportunities for early detection and intervention. Although most patients with advanced cancer receive treatment such as surgery, systemic chemotherapy, and some progress has been made in anti-cancer drugs, cell therapy, etc., pancreatic cancer is still a refractory disease with a low response rate to conventional immunochemotherapy [15,16]. In recent years, advancements and innovations in EUS technologies have markedly improved the sensitivity and accuracy of pancreatic cancer diagnosis [17,18]. Physicians are able to offer the treatment of PDAC complications via EUS as an alternative based on surgery or interventional radiology [19]. The development of EUS-guided fine-needle aspiration (EUS-FNA) has improved the diagnostic rate of pancreatic cancer [20]. As technology and endoscopic techniques continue to advance, EUS has emerged as a crucial modality in the diagnosis and management of pancreatic cancer. This review focuses on the role of endoscopy in the early screening, diagnosis, and treatment of pancreatic cancer.

## 2. EUS Screening and Detection

### 2.1. Screening of People at High Risk of Pancreatic Cancer

Pancreatic cancer starts insidiously, progresses rapidly, and is highly malignant. Therefore, screening for pancreatic cancer is necessary. EUS plays a pivotal role in the early detection of pancreatic cancer, owing to its high resolution and exceptional sensitivity in identifying pancreatic lesions. A multicenter study [21] conducted in the United States assessed 216 asymptomatic individuals at high risk of pancreatic cancer using both magnetic resonance imaging (MRI) and EUS. The results showed pancreatic abnormalities in 33.3% of cases via MRI and 42.6% via EUS, highlighting the critical role of EUS in screening high-risk populations. Regular EUS screenings can significantly improve the early diagnosis of pancreatic cancer. Despite this, early detection of sporadic pancreatic cancer is often overlooked in many cases. Targeted screening strategies for individuals with known risk factors have the potential to improve early detection rates, enhancing diagnostic effectiveness and patient outcomes [11]. Pancreatic cancer screening guidelines recommend that individuals at high risk of developing pancreatic cancer undergo regular screening through EUS and MRI. Additionally, annual screening for newly diagnosed diabetes is recommended as part of the risk assessment protocol [22]. Furthermore, pancreatic cancer risk models that incorporate multiple risk factors serve as valuable tools for early detection and diagnosis. These models can significantly improve the efficiency of pancreatic cancer screening efforts [23,24]. In future work, risk models can be further developed, and public education can be greatly strengthened to improve the awareness of early symptoms of pancreatic cancer so that medical help can be sought earlier.

### 2.2. EUS Combined with New Technologies

EUS-elastography (EUS-EG), which combines Endoscopic ultrasound and elastography techniques, is able to noninvasively evaluate the hardness characteristics of pancreatic tissue. Studies have shown that EUS-EG has high sensitivity and specificity in differentiating pancreatic cancer from pseudoneoplastic chronic pancreatitis [25]. This method is promising, but faces challenges such as standardization and establishing clear diagnostic thresholds [26]. Future research should focus on optimizing the application criteria of this technology to further improve its diagnostic efficiency.

Artificial intelligence (AI) has emerged as a powerful tool to enhance the accuracy of solid tumor screening. By facilitating the identification of high-risk populations, stratifying the risk associated with precancerous lesions, and predicting the progression of intraductal papillary mucinous neoplasms (IPMNs) to adenocarcinoma, AI exhibits significant potential in improving diagnostic precision for pancreatic cancer [27]. The latest Modified Faster R-CNN (M-F-RCNN) model, an AI algorithm model using EUS images to assist in the diagnosis of pancreatic cancer, has a sensitivity, specificity, and accuracy of 91.7%, 91.5%, and 91.6%, respectively [28]. However, the model currently processes fewer images and needs further verification. To maximize the effectiveness of EUS, it is crucial to integrate it within a broader clinical framework. This includes combining it with complementary diagnostic methods while considering factors such as the patient’s medical condition, financial constraints, and adherence to medical recommendations. Adopting this holistic and integrated approach is vital to ensure the practical and effective use of EUS in routine clinical practice.

## 3. Role of EUS in Pancreatic Cancer Diagnosis

### 3.1. High-Resolution Imaging and Diagnostic Capabilities of EUS

EUS enables the detection of small solid lesions and reliably facilitates cytopathological analysis through tissue sampling [10,29]. EUS provides high-resolution, detail-rich imaging of the pancreas, serving as a pivotal tool in the diagnosis of pancreatic cancer [30]. EUS surpasses computed tomography (CT) and MRI in accuracy for detecting small pancreatic cancers, with the capability to identify pancreatic masses as small as 5 mm in diameter [31]. Furthermore, EUS enables precise assessment of tumor infiltration depth, extent, and lymph node metastasis, thereby facilitating the accurate staging of pancreatic cancer. Concordance rates for determining the T stage and N stage using EUS are reported at 81.45% and 72.58%, respectively [32]. However, conventional EUS has certain limitations in distinguishing pancreatic cancer from other types of lesions, necessitating the development of supplementary diagnostic techniques to improve accuracy and reliability. In addition, at present, there are very few large-scale randomized controlled trials of EUS in the diagnosis and treatment of pancreatic cancer at home and abroad. Future research and development should be committed to conducting controlled trials to verify the effectiveness of EUS and promote the clinical and evidence-based medicine development of EUS in the field of pancreatic cancer.

### 3.2. EUS-FNA

EUS-FNA is a minimally invasive procedure that facilitates the collection of small tissue samples from lesions, allowing for pathological examination. Compared to traditional surgical biopsy, EUS-FNA is simpler, safer, and associated with fewer complications [33,34], with a complication rate of less than 1% [35]. A retrospective study reported that EUS-FNA demonstrated an overall sensitivity of 94.8%, specificity of 98.6%, and diagnostic accuracy of 95.1% [33]. In recent years, EUS-FNA has gained widespread application in the diagnosis of various diseases, including pancreatic disorders, with numerous studies substantiating its accuracy and safety. For instance, one study demonstrated that EUS-FNA achieved a sample adequacy rate of 96.8% and a diagnostic sensitivity of 60.2% in the evaluation of pancreatic tumors. These findings highlight the significant advantages of EUS-FNA in pathological assessment [20]. Various gene, nucleic acid, and protein analyses have been developed based on histological or cytological specimens obtained through EUS-FNA. These advancements aid in refining the diagnosis, identifying the biological characteristics of the patient, and facilitating the implementation of personalized medicine [36,37,38].

### 3.3. Emerging Technologies Complementing EUS-FNA

When conventional cytopathology fails to yield a definitive diagnosis, an advanced optical system, spatial-domain low-coherence quantitative phase microscopy (SL-QPM), can be utilized as an alternative diagnostic tool. This technology significantly enhances the diagnostic accuracy of cytological analysis in EUS-FNA, improving the sensitivity for detecting pancreatic cancer from 72% to 94% [39]. However, EUS-FNA carries a certain risk of false-negative results [40]. In such cases, contrast-enhanced endoscopic ultrasonography (CEH-EUS) can be employed for further diagnostic evaluation [41]. CEH-EUS achieves a sensitivity of up to 93% and a specificity of 80% in the diagnosis of pancreatic cancer [42]. Compared to traditional EUS, CEH-EUS offers superior sensitivity and specificity, enhancing its diagnostic efficacy [43]. In addition, CEH-EUS is utilized to evaluate the therapeutic response of pancreatic cancer to chemotherapy and to assess the prognosis of both pancreatic cancer and pancreatic neuroendocrine tumors [44]. The application of CEH-EUS in the process of the diagnosis of solid pancreatic tumors remains an area that warrants further investigation and exploration.

### 3.4. EUS-FNB: An Evolution of EUS-FNA

With the introduction of new needles, the EUS-guided fine-needle biopsy (EUS-FNB) technology, which is based on FNA with modifications, has gradually emerged. The analysis by Li et al. [45] demonstrated that EUS-FNB provides superior sampling quality, higher diagnostic accuracy, and requires fewer needle passes compared to EUS-FNA. A separate meta-analysis indicated that while EUS-FNA and EUS-FNB show similar diagnostic accuracy and sample adequacy, EUS-FNB provides the benefit of obtaining a diagnosis with fewer needle passes [46]. Furthermore, no notable differences have been found between EUS-FNA and EUS-FNB regarding complication or technical success rates [45].

### 3.5. Innovative Real-Time Imaging with EUS-nCLE

EUS-needle-based confocal laser endomicroscopy (EUS-nCLE) is an innovative technology that provides real-time imaging, offering a novel approach to diagnostic visualization. Konda et al. [47] were the first to establish the feasibility of EUS-nCLE; however, the procedure is associated with a potential risk of serious adverse events. In a prospective single-blind study, Kongkam et al. [48] were the first to assess the effectiveness of EUS-nCLE in differentiating between benign and malignant solid lesions of the pancreas. The results demonstrated a diagnostic accuracy of 90.9%, with only one case of bleeding reported. This study validated the feasibility and safety of EUS-nCLE in the differential diagnosis of solid pancreatic lesions.

### 3.6. Combine Other Technologies

EUS combined with other diagnostic methods can improve the sensitivity and accuracy of pancreatic cancer diagnosis [49]. Studies have shown that the accuracy, sensitivity, specificity, positive predictive value, and negative predictive value of EUS combined with abdominal CT/MRI in the diagnosis of pancreatic cancer are 98.78%, 97.62%, 100.00%, 100.00%, and 97.56%, all of which are higher than that of EUS and CT/MRI alone. EUS combined with CT/MRI can improve the detection rate of pancreatic cancer, and has certain significance for early diagnosis and treatment of pancreatic cancer [50]. In a study to evaluate the diagnostic value of EUS combined with enhanced CT in the clinical staging of pancreatic cancer, 80 patients with suspected pancreatic cancer were examined by EUS and enhanced CT, with pathological findings as the gold standard. The results showed that the sensitivity (97.18%) and accuracy (96.25%) of EUS combined with enhanced CT were significantly higher than those of EUS or enhanced CT alone. The accuracy of combined diagnosis in T2, T3, and T4 staging was also better than that of single examination, indicating that EUS combined with enhanced CT has higher efficacy and staging accuracy in the diagnosis of pancreatic cancer [49]. A preoperative imaging study of 181 patients undergoing surgery for left pancreatic lesions showed sensitivity of 71% for both CT and EUS-FNA/B. When EUS was combined with FNA/B, the sensitivity increased from 64% to 71% [51]. A number of articles have shown that EUS combined with other techniques can improve diagnostic capability [52]. In addition, endoscopic retrograde cholangiopancreatography (ERCP) continues to play an indispensable role in the diagnosis and management of pancreatic diseases. ERCP-mediated cytological examination achieves a detection rate of 72.2% in stage 0 pancreatic cancer patients, compared to a detection rate of only 16.7% with EUS-FNA [18]. Unfortunately, pancreatic juice cytology can be influenced by factors such as the location and size of the duct, and patients frequently experience complications such as pancreatitis following the procedure [53]. As a result, ERCP is more commonly employed for therapeutic purposes.

EUS plays a pivotal role in the diagnosis of pancreatic space-occupying lesions, offering critical insights into their characterization and management. CEH-EUS enhances the characterization of pancreatic lesions, facilitates their differential diagnosis, and aids in identifying optimal target sites for fine-needle aspiration or biopsy procedures. EUS-FNA and EUS-FNB enable pathological diagnosis by obtaining tissue samples, allowing for precise determination of the nature of pancreatic lesions. Meanwhile, the emerging EUS-nCLE technology offers real-time optical biopsy capabilities under endoscopic ultrasound, demonstrating promising potential for clinical application. Despite significant advancements in imaging techniques and diagnostic technologies, the early detection of pancreatic cancer continues to pose a formidable challenge [29]. In addition, EUS still has many potential limitations (such as technical dependence and difficulty of operation, limitation of lesion identification, limitation of scope and depth of examination, and influence of patient factors) [54]. We can make improvements by improving technology and equipment, enhancing operator training, and optimizing patient selection and examination processes. It is anticipated that more diagnostic measures will emerge in the future to address these limitations.

## 4. EUS-Guided Palliative Treatment for Pancreatic Cancer

**Figure 1:**
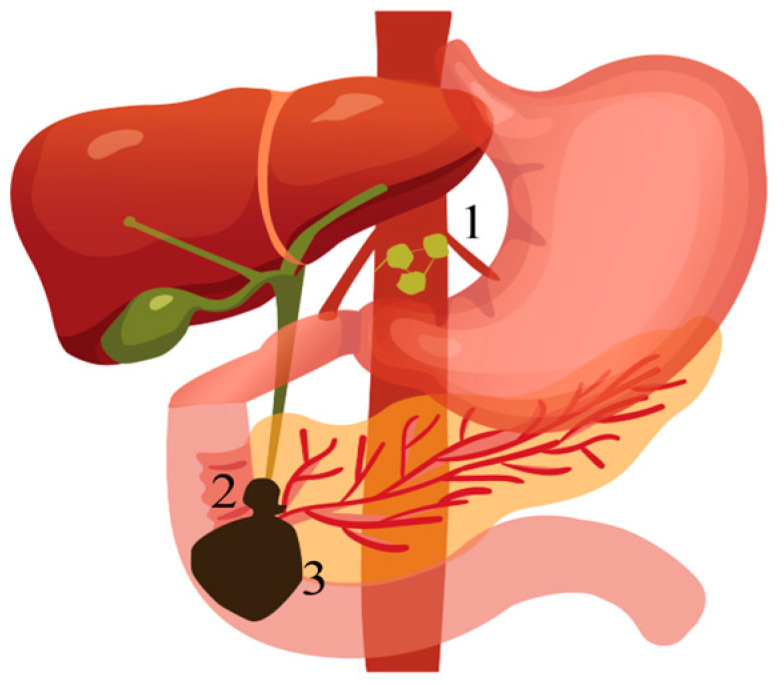
The targets of pancreatic cancer complications (position is often involved): (**1**) The celiac plexus is the target for pain treatment. (**2**) Advanced tumors of the biliary tract and duodenum are sites of progression. (**3**) The descending part of the duodenum is the common site of gastric outlet obstruction.

Clinical manifestations of pancreatic cancer commonly include painless jaundice and abdominal or back pain. However, the diagnosis is often complicated by the presence of nonspecific symptoms such as fatigue, unintended weight loss, and anorexia, which lack specificity and may delay recognition of the disease [55]. Less frequently observed clinical manifestations of pancreatic cancer include steatorrhea resulting from malignant obstruction of the main pancreatic duct, acute pancreatitis, gastric outlet obstruction, and venous thromboembolism. The complications associated with pancreatic cancer are influenced by the primary tumor’s anatomical location and the extent of disease progression (see Figure 1). These complications commonly include biliary obstruction, gastric outlet obstruction, and pain [19] (see Table 1).

### 4.1. Malignant Biliary Obstruction

Painless jaundice caused by biliary obstruction is considered one of the most common symptoms in patients with pancreatic head ductal adenocarcinoma, occurring in approximately three-quarters of this population [67]. Pancreatitis resulting from biliary obstruction is among the most frequent clinical manifestations of pancreatic cancer involving the pancreatic head [68]. The obstruction primarily arises from external compression of the bile duct by the tumor or the intraluminal growth of the malignant lesion [55]. Currently, ERCP combined with biliary stent placement has emerged as the first-line therapeutic approach for managing biliary obstruction [69]. ERCP is regarded as the gold standard for the management and relief of biliary obstruction [29]. The ERCP trans-papillary approach is an important milestone in the treatment of biliary obstruction and has the advantage of avoiding external drainage, reducing mortality, shortening hospital stay, and decreasing the incidence of adverse events compared to percutaneous transhepatic biliary drainage (PTBD) [70,71,72]. Compared to percutaneous and surgical biliary decompression, ERCP is associated with reduced morbidity and mortality, fewer complications, and significant improvements in patient quality of life [73]. Moreover, when compared to surgical methods, ERCP demonstrates lower rates of morbidity, intraoperative and postoperative complications, as well as 30-day mortality [74]. However, hepaticojejunostomy has been shown to reduce the incidence of recurrent jaundice [75]. Inamdar et al. found that biliary drainage via ERCP is linked to a lower incidence of complications and shorter hospital stays compared to PTBD [70]. Furthermore, a propensity score-matched analysis revealed that patients undergoing PTBD exhibited lower postoperative survival rates and an increased risk of metastasis compared to those treated with ERCP [76]. When a patient’s gastric or duodenal anatomy is altered, rendering ERCP unable to access the bile duct requiring drainage, or when ERCP fails to achieve sufficient biliary drainage, PTBD is the first-line alternative [77].

When ERCP fails, EUS-guided biliary access and stent placement have been widely adopted as a reliable alternative. This method has demonstrated steadily increasing technical and clinical success rates over time [71,78]. The use of perioperative ERCP for biliary drainage in cases of resectable pancreatic cancer remains a subject of ongoing debate. Routine postoperative biliary drainage is generally not recommended, as ERCP may delay surgical intervention or complicate the procedure [79]. Studies have shown that preoperative biliary drainage can improve survival rates in appropriately selected cases [79]. For patients who may require delayed surgical resection or neoadjuvant chemoradiotherapy, plastic stents are typically placed initially in cases of cholangitis or biliary obstruction. However, covered metal stents have demonstrated superior patency compared to alternative options and are typically employed following the confirmation of a definitive diagnosis and the establishment of a treatment plan [29]. Since Giovannini et al. [80] first demonstrated the feasibility of EUS-guided biliary drainage (EUS-BD) in 2001, this technique has emerged as a viable and effective alternative to PTBD. It is now commonly utilized as a salvage procedure in cases where ERCP is unsuccessful [81,82].

**Figure 2:**
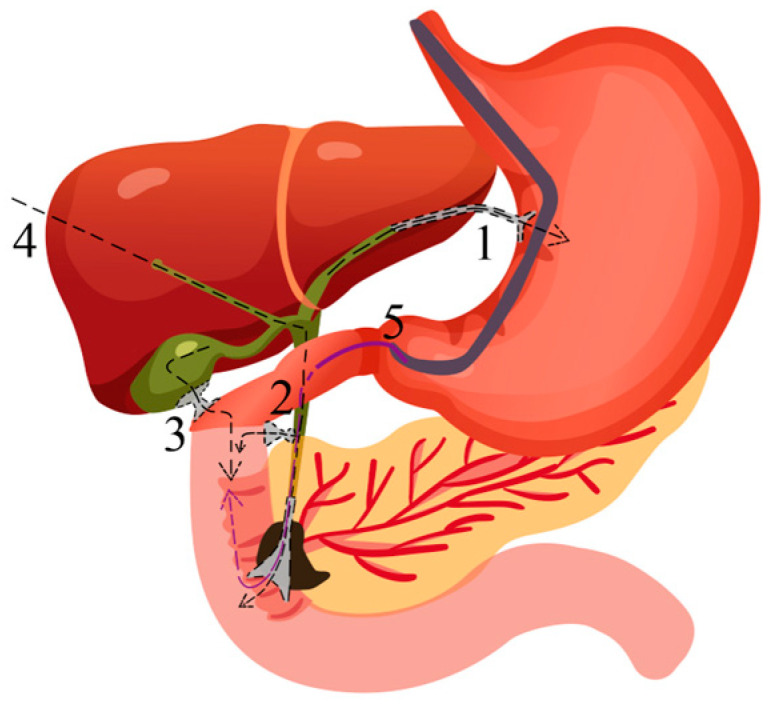
EUS-BD approaches for malignant biliary obstruction (the dashed line is the drainage route): (**1**) EUS-HGS, (**2**) EUS-CDS, and (**3**) EUS-GBD are classified as transluminal drainage procedures; (**4**) EUS-AG; (**5**) EUS-RV.

MBO is categorized according to its anatomical location into distal malignant biliary obstruction (dMBO) and proximal malignant biliary obstruction (pMBO). DMBO typically results from malignant involvement of the distal common bile duct (CBD), often caused by internal or external compression due to conditions such as pancreatic head cancer, cholangiocarcinoma, ampullary cancer, or metastatic lymph node invasion [83,84]. PMBO refers to the malignant involvement of the proximal CBD, resulting from intraluminal tumor obstruction or external compression. PMBO frequently affects the junctions of the hepatic ducts and can progress to hilar malignant biliary obstruction (hMBO) [85]. PMBO is commonly seen in cholangiocarcinoma. Endoscopic therapy has traditionally been centered around ERCP. However, as shown by recent studies and ongoing trials, EUS-guided approaches are increasingly regarded as the primary alternative treatment when ERCP fails. Notably, the incidence of post-ERCP pancreatitis (PEP) is markedly higher compared to that observed with EUS-BD [86]. EUS-BD serves as an effective alternative in situations where ERCP fails, biliary cannulation is challenging, or anatomical alterations following surgery preclude conventional approaches [71]. EUS-BD can be broadly categorized into three main approaches: rendezvous, antegrade, and transmural stenting [81] (see Figure 2). EUS-BD includes EUS-guided choledochoduodenostomy (EUS-CDS), EUS-guided hepaticogastrostomy (EUS-HGS), EUS-guided gallbladder drainage (EUS-GBD), EUS-guided antegrade stenting (EUS-AG), and EUS-guided rendezvous (EUS-RV). Studies have shown that EUS-BD is a minimally invasive option associated with fewer procedure-related adverse events and a lower rate of reintervention [87]. The meta-analysis conducted by Sharaiha et al. further validated the findings of the study, providing robust evidence to support its conclusions [60].

EUS-CDS is regarded as the preferred therapeutic approach for patients with dMBO when ERCP fails [88]. EUS-CDS entails the insertion of a stent from the duodenum, generally at the duodenal bulb, into the common bile duct, thereby facilitating bile drainage. Compared to ERCP, EUS-CDS provides distinct advantages by bypassing the major papilla and pancreatic duct, thereby eliminating the risk of postprocedural pancreatitis. Furthermore, the stent in EUS-CDS is typically placed proximal to the obstruction, effectively reducing the risk of stent occlusion due to tumor overgrowth. These studies have shown no significant differences between EUS-CDS and ERCP in terms of technical and clinical success rates [61,89]. However, compared to ERCP, EUS-CDS is associated with a lower incidence of adverse events (notably the absence of postoperative pancreatitis) and a reduced rate of reintervention [90].

When tumor invasion of the duodenum obstructs access to the major papilla or the duodenal bulb, EUS-HGS serves as an alternative approach for biliary drainage. EUS-HGS allows the formation of a fistula between the gastric wall and the left intrahepatic bile duct, positioning it as the preferred method for managing hMBO [19,77]. Additionally, in cases where ERCP and PTBD fail to achieve adequate biliary drainage, the European Society of Gastrointestinal Endoscopy (ESGE) recommends that EUS-HGS be reserved for patients with inoperable malignant hilar biliary obstruction associated with left hepatic duct dilation [91].

The initial steps of EUS-AG closely resemble those of EUS-HGS, and if the lead fails to pass through the nipple, the surgery can be converted to EUS-HGS as long as the puncture point in the lumen is the proximal stomach. EUS-AG has recently been reported to have an overall clinical success rate of 97.2% and a mortality rate of 0%, with pancreatitis being the most common adverse event [92]. EUS-RV is a technique that employs EUS for image-guided biliary access, achieved by puncturing the intrahepatic bile duct through the stomach or the common bile duct through the duodenum. Next, a guidewire is placed across the biliary orifice or papilla under fluoroscopic guidance to facilitate the subsequent ERCP procedure. EUS-RV can be utilized for the evaluation of both malignant and benign conditions. Adverse events linked to EUS-RV have been reported to include abdominal pain, peritonitis, bile leakage, pneumoperitoneum, and pancreatitis [93]. EUS-GBD is commonly employed in non-operative patients with acute cholecystitis. Associated adverse events include bleeding, bile leakage, perforation, stent occlusion, and stent displacement [94]. However, there are few clinical studies and trials of EUS-AG, EUS-RV, and EUS-GBD on palliative treatment in malignant biliary drainage of pancreatic cancer, and more trials are expected to prove it in the future.

Moreover, in the presence of gastroduodenal stents, EUS-guided approaches have been shown to be technically and clinically superior to ERCP [95], particularly in cases involving double obstruction [96]. The application of EUS technology in biliary drainage represents a transformative shift from conventional treatment modalities to precise, minimally invasive interventions. As this technology matures, its utility in managing biliary obstructions, particularly in complex cases, is anticipated to become increasingly significant. The selection of specific EUS-guided techniques should be carefully tailored to individual patient factors, including anatomical variations, tumor location, and the feasibility of surgical intervention. EUS-guided biliary and pancreatic drainage procedures are technically demanding and carry a relatively high risk of adverse events, necessitating their performance by highly skilled and experienced endoscopists. The absence of standardized techniques poses a significant barrier to the widespread clinical adoption of EUS for biliary drainage. To address these challenges, future efforts should prioritize large-scale randomized controlled trials to comprehensively evaluate the long-term efficacy and safety of EUS technologies. The development of innovative stents and devices is also critical to reducing complication rates and enhancing therapeutic outcomes. Moreover, integrating artificial intelligence (AI) and advanced imaging analysis into EUS procedures holds considerable promise for improving diagnostic accuracy and procedural success, paving the way for broader and more effective clinical applications.

### 4.2. Gastric Outlet Obstruction

GOO is a clinical syndrome marked by symptoms including postprandial vomiting, abdominal pain, and abdominal distension, often progressing to significant weight loss in advanced stages due to insufficient oral intake [97]. GOO occurs in approximately 15–20% of patients with pancreatic cancer, profoundly affecting their quality of life [98]. Palliative care strategies such as surgery (bypass), endoscopy, or radiotherapy can be chosen. In the past, patients with GOO who lacked minimally invasive treatment options often underwent surgical bypass to alleviate gastrointestinal obstruction. However, individuals with advanced gastrointestinal disease are often in poor condition, rendering them unsuitable for surgery due to its associated risks [77]. Furthermore, patients with gastric lymphoma face a high risk of chemotherapy-induced gastric perforation [97,99], underscoring the urgent need for the development of less invasive treatment alternatives.

Enteral stent placement remains the primary endoscopic intervention for the management of malignant gastric outlet obstruction (mGOO). For patients with malignant gastrointestinal obstruction who are ineligible for surgery and have a limited life expectancy, enteral stenting provides a viable option to restore luminal patency [100,101]. However, compared to gastrojejunostomy, enteral stenting has a shorter duration of patency and is associated with shorter patient survival [102].

Since Fritscher-Ravens et al. [103] first hypothesized and tested EUS-guided gastroenterostomy (EUS-GE) in an animal model in 2002, this technique has steadily advanced. The introduction of lumen-apposing metal stents (LAMSs) has greatly simplified the EUS-GE procedure. EUS-GE involves the placement of a LAMS, which facilitates strong luminal apposition and creates a fistulous connection between the stomach and jejunum. With advancements in technology, EUS-GE has been proven to be a safe and effective procedure, demonstrating high clinical and technical success rates [104,105]. It is linked to a low incidence of adverse events, shorter procedure durations, and decreased hospital stays [62,106]. Consequently, EUS-GE has been proposed as a viable alternative to conventional enteral stent placement [107].

Furthermore, gastrojejunostomy (GJ) performed using a hybrid natural orifice transluminal endoscopic surgery (NOTES) technique has emerged as an innovative alternative within the spectrum of endoscopic treatment options. Trials conducted on porcine models have demonstrated that transluminal endoscopic gastrojejunostomy (TEGJ) is technically feasible [108]. However, this procedure remains under development [77].

In summary, EUS-GE, enteral stent placement, and surgical intervention each represent effective palliative treatment modalities for mGOO. The selection of treatment should be individualized based on the patient’s specific clinical characteristics to optimize outcomes and enhance success rates. With the advancement of new technologies, optimizing procedural workflows, improving device design, and establishing standardized guidelines have become critical priorities. The widespread adoption of techniques such as EUS-GE requires robust clinical evidence from high-quality studies. Future research should focus on tailoring treatment strategies to individual patients, particularly those with poor surgical candidacy or complex symptoms, to identify the most suitable therapeutic approach. The integration of endoscopic treatments with chemoradiotherapy holds promise for improving patient outcomes, offering a more comprehensive approach to management. Additionally, further exploration and development of innovative techniques such as TEGJ could potentially revolutionize the treatment landscape for GOO, providing new and effective solutions for these challenging cases.

### 4.3. Pain

Pain management is a significant challenge in the treatment of pancreatic cancer. At diagnosis, 70–90% of patients with advanced biliary and pancreatic cancers experience pain, often resulting from neural invasion by tumor cells [109]. Studies have shown that pain is related with reduced survival rates [110]. Approximately half of the patients require opioid medications for pain relief [110]. However, prolonged administration of high-dose opioids is associated with the development of tolerance, and patients may be at risk of opioid dependence or addiction [111]. For patients with pain refractory to oral therapies, EUS-guided celiac plexus neurolysis (EUS-CPN) represents a viable therapeutic option. EUS-CPN has been demonstrated to significantly alleviate chronic pain in pancreatic cancer patients, thereby reducing their reliance on potent analgesics [77].

Pancreatic cancer frequently infiltrates the retroperitoneal nerve plexus. EUS-CPN alleviates pain by injecting neurolytic agents, such as absolute ethanol or bupivacaine, into the celiac ganglia. A meta-analysis demonstrated that EUS-CPN significantly reduces pain in patients with pancreatic cancer (risk ratio [RR] = 0.83, 95% confidence interval [CI]: 0.83–0.83) and achieves complete pain relief in a subset of cases (RR = 0.09, 95% CI: 0.09–0.09) [112]. The adverse effects of EUS-CPN are typically mild and include transient diarrhea and postoperative hypotension [19]. Adverse events linked to EUS-CPN primarily involve complications like reversible or irreversible paralysis, organ damage, and gastric necrosis [29]. Yoon et al. compared EUS-CPN with traditional percutaneous celiac neurolysis (PCN) and found that EUS-CPN and PCN exhibit similar efficacy and safety in managing refractory pain in pancreatic cancer patients [113].

A randomized trial comparing EUS-CPN with EUS-guided radiofrequency ablation (EUS-RFA) demonstrated that EUS-RFA offered superior pain relief for patients compared to EUS-CPN [114]. EUS-RFA is a minimally invasive, simple, and safe technique for treating localized tumors. It has been shown to effectively alleviate pain in patients [115]. A prospective trial demonstrated that EUS-RFA is safe, well-tolerated, and can be performed concurrently with standard chemotherapy [116].

Sakamoto et al. [117] first described an alternative method potentially more suitable for patients with advanced abdominal cancers: EUS-guided broad plexus neurolysis (EUS-BPN). Compared to traditional EUS-CPN, BPN demonstrated superior pain relief scores at both 7 and 30 days, without causing severe complications. However, EUS-BPN currently lacks further clinical trials to substantiate its safety and efficacy.

Advancements in EUS technology have made it possible to visualize and access individual celiac ganglia, allowing for direct injection into these ganglia to perform celiac ganglia neurolysis (CGN) [118]. Unfortunately, current trials indicate that EUS-CGN does not improve the average duration of pain relief [119] and may even shorten survival time [120].

The advancement of techniques such as EUS-CPN, EUS-RFA, and EUS-BPN offers clinicians a broader array of options for pain management. Tailoring the choice of intervention based on the patient’s condition, pain severity, and expected survival can further optimize the effectiveness of pain control strategies. Additionally, further clinical trials of emerging technologies should focus on evaluating their long-term efficacy and safety. Concurrently, existing techniques should be refined by improving ablation devices and injection materials to reduce the risk of complications. Moving forward, advancements in technology and clinical research should aim to enhance pain management protocols, providing pancreatic cancer patients with safer, more effective, and personalized treatment options. Such progress will not only improve quality of life but also contribute to extending survival outcomes.

## 5. Future Research Directions for EUS in Pancreatic Cancer Treatment

In the future, research on EUS in pancreatic cancer treatment will primarily focus on advancing the technology and exploring novel therapeutic approaches. With the continuous upgrading of EUS equipment, emerging interventional techniques such as EUS-guided local drug injection and radiofrequency ablation are rapidly evolving. These advancements hold the potential to significantly enhance the therapeutic outcomes for pancreatic cancer [15]. EUS-guided fine-needle injection (EUS-FNI) is an emerging technique for enhancing localized intratumoral drug delivery. It is considered a potential approach for preoperative tumor debulking or as palliative treatment for unresectable tumors with mass effects [121]. In 2000, the first attempt to treat pancreatic ductal adenocarcinoma using EUS-FNI for immunotherapy with allogeneic mixed lymphocyte cultures was reported [122]. Although definitive evidence of EUS-FNI’s efficacy in large-scale trials is currently lacking, several reports have highlighted its potential benefits [15].

Later, large-scale trials should be improved to prove the efficacy of EUS-FNI, and CE-EUS and elastic imaging can also be used to improve tumor visualization and improve targeting accuracy, so as to improve the safety of EUS-FNI and other interventional techniques. In addition, the therapeutic regimen can be optimized, and new methods such as immunotherapy and targeted molecular therapy can be integrated into the EUS-guided therapeutic mode. In recent years, antitumor drug delivery of platelets has been studied intensively [123], and it is expected that they can bind to EUS in the future. In summary, the interventional capabilities of EUS are poised to remain integral to the evolving management strategies for pancreatic cancer. Future research will aim to refine current techniques and investigate new therapeutic approaches, with the goal of enhancing patient outcomes and quality of life.

## 6. Conclusions

EUS, as an emerging imaging modality, holds significant promise in the diagnosis and treatment of pancreatic cancer. Research has demonstrated that EUS not only provides high-resolution imaging of pancreatic tissue but also markedly enhances the early detection rate of pancreatic cancer. Its distinctive advantage lies in its ability to identify small lesions and facilitate precise pathological sampling. However, the long-term efficacy and safety of EUS in managing pancreatic cancer have yet to be fully substantiated by robust clinical evidence. Future investigations should prioritize large-scale, randomized controlled trials to comprehensively assess its effectiveness and potential risks. Such studies will aid in establishing more rigorous criteria for diagnosis and treatment, thereby equipping clinicians with reliable evidence for informed decision-making regarding therapeutic options. Moreover, integrating EUS with other imaging techniques such as CT and MRI may further enhance survival rates and quality of life for patients with pancreatic cancer. The application of multimodal imaging renders lesion evaluation more comprehensive while providing substantial support for developing personalized treatment plans—potentially paving new avenues for research into pancreatic cancer. Furthermore, EUS is not only significant in the diagnosis and treatment of PDAC but also plays an important role in the diagnosis and treatment of pancreatic neuroendocrine tumors (pNENs). Multiple studies have demonstrated the efficacy and safety of EUS in the treatment of pNENs [124,125]. However, current studies lack sufficient long-term efficacy data, and the number of pNEN patients is relatively small. More trials are anticipated in the future to further validate the role of EUS in the diagnosis and treatment of pNENs.

In conclusion, EUS possesses considerable potential in the realm of diagnosing and treating pancreatic cancer. Through ongoing research efforts and clinical practice, we aim to deepen our understanding of this technology’s capabilities within the context of pancreatic cancer management, ultimately leading to improved patient quality of life and prognosis.

## Figures and Tables

**Table 1:** Adverse effects of therapeutic EUS in pancreatic cancer.

Complications of Pancreatic Cancer	Endoscopic Ultrasonography	Complications of Operation	Reference
MBO	EUS-guided choledochoduodenostomy	Cholangitis or cholecystitis, pneumoperitoneum, peritonitis associated with bile leak, recurrent biliary obstruction	[56,57]
	EUS-guided hepaticogastrostomy	Bile leakage, recurrent biliary obstruction (RBO)	[58]
	EUS-guided antegrade stenting	Pancreatitis, abdominal pain, fever, cholangitis	[59]
	EUS-guided rendezvous	Abdominal pain, peritonitis, bile leakage, pneumoperitoneum, and pancreatitis	[60]
	EUS-guided gallbladder drainage	Bleeding, bile leakage, perforation, stent occlusion, stent displacement	[61]
GOO	EUS-guided gastroenterostomy	LAMS misdeployment, leakage at the site of the LAMS, LAMS mesh erosion, abdominal pain, bleeding, infection, gastric leak, stent ingrowth, stent failure	[62]
PAIN	EUS-guided celiac plexus neurolysis	The effects were short-lived and there were no serious adverse events	[63]
	EUS-guided radiofrequency ablation	Abdominal pain, no serious adverse events	[64]
	EUS-guided broad plexus neurolysis	No serious adverse events	[65]
	EUS-celiac ganglia neurolysis	Transient pain exacerbation, transient hypotension, transient diarrhea, and inebriation	[66]

## Data Availability

No new data were created or analyzed in this study. Data sharing is not applicable to this article.

## Abbreviations

The following abbreviations are used in this manuscript:

EUS	Endoscopic ultrasonography
MBO	Malignant biliary obstruction
GOO	Gastric outlet obstruction
HDL	high-density lipoprotein
PDAC	Pancreatic ductal adenocarcinoma
EUS-FNA	EUS-guided fine-needle aspiration
MRI	Magnetic resonance imaging
EUS-EG	EUS-elastography
AI	Artificial intelligence
IPMN	Intraductal papillary mucinous neoplasms
M-F-RCNN	Modified Faster R-CNN
CT	Computed tomography
SL-QPM	Spatial-domain low-coherence quantitative phase microscopy
CEH-EUS	Contrast-enhanced endoscopic ultrasonography
EUS-FNB	EUS-guided fine-needle biopsy
EUS-nCLE	EUS-needle-based confocal laser endomicroscopy
PTBD	Percutaneous transhepatic biliary drainage
EUS-BD	EUS-guided biliary drainage
dMBO	Distal malignant biliary obstruction
pMBO	Proximal malignant biliary obstruction
hMBO	Hilar malignant biliary obstruction
EUS-CDS	EUS-guided choledochoduodenostomy
EUS-HGS	EUS-guided hepaticogastrostomy
EUS-GBD	EUS-guided gallbladder drainage
EUS-AG	EUS-guided antegrade stenting
EUS-RV	EUS-guided rendezvous
EUS-GE	EUS-guided gastroenterostomy
NOTES	Natural orifice transluminal endoscopic surgery
TEGJ	Transluminal endoscopic gastrojejunostomy
EUS-CPN	EUS-guided celiac plexus neurolysis
PCN	Percutaneous celiac neurolysis
EUS-BPN	EUS-guided broad plexus neurolysis
CGN	Celiac ganglia neurolysis
EUS-FNI	EUS-guided fine-needle injection
pNENs	Pancreatic neuroendocrine tumors
EUS-RFA	EUS-guided radiofrequency ablation
